# Identification of novel split-GAL4 drivers for the characterization of enteroendocrine cells in the *Drosophila melanogaster* midgut

**DOI:** 10.1093/g3journal/jkac102

**Published:** 2022-04-29

**Authors:** Jessica M Holsopple, Kevin R Cook, Ellen M Popodi

**Affiliations:** Department of Biology, Bloomington Drosophila Stock Center, Indiana University, Bloomington, IN 47405, USA

**Keywords:** Drosophila, intestine, gut, midgut, split-GAL4, endocrine, enteroendocrine cell

## Abstract

The *Drosophila melanogaster* midgut is commonly studied as a model epithelial tissue for many reasons, one of which is the presence of a diverse population of secretory cells called enteroendocrine cells. Subpopulations of these cells secrete various combinations of peptide hormones which have systemic effects on the organism. Many of these hormones are also produced in the Drosophila brain. The split-GAL4 system has been useful for identifying and manipulating discrete groups of cells, but previously characterized split-GAL4 drivers have not driven expression in high proportions of enteroendocrine cells. In this study, we screened candidate split-GAL4 drivers for enteroendocrine cell expression using known reference drivers for this cell type and discovered a new split-GAL4 driver pair that confers expression in a greater number of enteroendocrine cells than previously characterized driver pairs. The new pair demonstrates less brain expression, thereby providing better tools for disentangling the physiological roles of gut- and brain-secreted peptides. We also identified additional split-GAL4 drivers that promote expression in discrete subpopulations of enteroendocrine cells. Overall, the tools reported here will help researchers better target enteroendocrine cell subpopulations.

## Introduction 

The *Drosophila melanogaster* intestine is a widely studied model tissue due to its many parallels to mammalian digestive systems, the extensive genetic manipulations available in Drosophila, and the simplicity of the tissue relative to the digestive tracts of other organisms (Zwick *et al.* 2019). The Drosophila intestine has 3 distinct regions: the foregut, midgut, and hindgut. Of these regions, the midgut is the most commonly studied. The midgut epithelium is made up of 4 cell types: intestinal stem cells (ISCs), their differentiating daughters called enteroblasts (EBs) (together termed progenitor cells), and 2 terminally differentiated cell types consisting of absorptive enterocytes (ECs) and secretory enteroendocrine cells (EEs) (Ohlstein and Spradling 2006; Miguel-Aliaga *et al.* 2018). Despite the relatively uncomplicated nature of this tissue, it provides functions similar to those of the mammalian stomach and small intestine.

As an important interface with the environment, the intestine must have the ability to adapt to rapidly changing conditions, such as feeding, starvation, and ingestion of toxins or pathogenic bacteria. To coordinate these adaptations, the Drosophila midgut uses diverse combinations of hormone secretions from EEs to communicate with cells both within the midgut and in other body regions (Veenstra *et al.* 2008; Veenstra and Ida 2014). RNAseq studies have shown that single EE cells can secrete combinations of up to 5 different peptide hormones, a fact that lends itself to categorizing subpopulations of EEs based on the hormones secreted (Guo *et al.* 2019). Hormone secretion patterns are often regionalized, with subsets of EEs appearing in distinct portions of the intestine (Veenstra *et al.* 2008; Veenstra and Ida 2014; Guo *et al.* 2019; Hung *et al.* 2020). To date, there are few reagents available to manipulate discrete subpopulations of EEs.

The UAS–GAL4 system is a powerful tool of modern Drosophila research but GAL4 drivers unfortunately often lack the specificity needed to identify and manipulate small groups of cells, such as specific EE subtypes. To increase resolution, researchers have embraced the split-GAL4 system, in which expression of the GAL4 activation domain (AD) is controlled by different regulatory sequences than expression of the GAL4 DNA-binding domain (DBD). When both the AD and the DBD are expressed in the same cell, these domains can bind via attached leucine zipper tags to reconstitute functional GAL4, which can then activate expression of *UAS* transgenes (Luan *et al.* 2006). Because functional GAL4 is produced only when both domains are expressed together, this system allows for more fine-tuned patterns of *UAS* activation than the original UAS–GAL4 system. In this study, drivers contained the activation domain from the *p65* gene (*p65AD*) in place of GAL4AD because p65AD induces stronger transcriptional activation (Dionne *et al.* 2018).

To improve the utility of the split-GAL4 system, recent work characterized the expression patterns of 7,304 experimental split-GAL4 drivers in the Drosophila midgut by both cell type and region via 2 distinct screens (Ariyapala *et al.* 2020). First, experimental AD or DBD split-GAL4 drivers were combined with DBD or AD reference drivers known to direct expression only in the intestinal epithelium. Experimental drivers that did not direct expression in the midgut were not screened further. Drivers that did direct midgut expression were combined with appropriate AD or DBD reference drivers specific to each of the major midgut cell types (progenitor cells, ECs, or EEs) in a second screen to determine the unique cell-type expression profiles of each experimental split-GAL4 driver. The expression profiles of the intestine-specific reference drivers used in the first screen allowed for detection of high percentages of total midgut ISCs, EBs, and ECs, but a much lower percentage of EEs. Similarly, the reference drivers used in the second screen for recognition of progenitor cells and ECs enabled detection of a high percentage of their respective cell types, while the EE reference drivers facilitated detection of only half of EEs. These reference drivers were useful in characterizing the expression patterns of experimental drivers, but their weaknesses with respect to EEs suggest that many split-GAL4 drivers directing expression in EEs were overlooked by these screens. Reference drivers expressing in a higher proportion of EEs are needed to advance the study of this important cell type.

In Drosophila, a subset of neuropeptides produced in the brain are also produced by midgut EE cells (Nässel and Winther 2010). For studies of neuropeptide signaling, it would be advantageous for split-GAL4 reference drivers expressed in EEs to be characterized in the brain. Ideally, drivers could be identified that allow the experimental manipulation of neuropeptide secretion specifically in EEs.

Here, we report the identification of a more sensitive and specific pair of EE reference drivers. The AD and DBD drivers share the same enhancer fragment, and we used them to identify and characterize 43 additional EE drivers that had been missed in previous screens. Our preliminary analysis suggests that the new driver pair activates less *UAS* reporter expression in the brain than the previously used EE reference drivers, which should prove beneficial to future studies of neuropeptide-related physiology.

## Materials and methods

### Drosophila strains and husbandry

Fly strains were cultured at 25° on standard Bloomington media (https://bdsc.indiana.edu/information/recipes/bloomfood.html). The genotypes of all starting stocks used in this study are listed in Supplementary Table 1. The *PBac{UAS-DSCP-6XEGFP}VK00018* strain used for reporter comparisons was a gift from Steve Stowers (Montana State University).

### Dissections and immunostaining

To analyze the fluorescence from *UAS-Stinger* and *lexAop-tdTomato*, adult gastrointestinal tracts were dissected, fixed, and counterstained with DAPI as described in Ariyapala *et al.* (2020). Intestines from selected adults were antibody stained as described in Ariyapala *et al.* (2020) using primary mouse anti-Prospero (Pros) (MR1A, 1:100; Developmental Studies Hybridoma Bank) or rabbit anti-GFP (A-11122, Life Technologies, 1:1,000) antibody, and secondary AlexaFluor 568-conjugated goat anti-mouse or AlexaFluor 488-conjugated goat anti-rabbit antibody (A-11034 1:1,000; Life Technologies). In some cases, rhodamine-conjugated goat anti-Horseradish Peroxidase (HRP) antibody (123-025-021, 1:500; Jackson ImmunoResearch) was included during secondary antibody staining. When only anti-HRP was used, intestines were dissected and fixed as described in Ariyapala *et al.* (2020), washed 3 times in PBT [0.1% Triton X-100 in PBS (137 mM NaCl, 2.68 mM KCl, 10.1 mM Na_2_HPO_4_, and 1.76 mM KH_2_PO_4_, pH 7.4)], blocked in 0.5% bovine serum albumin and 5% normal goat serum in PBT for 1 h, incubated with DAPI and anti-HRP overnight, washed 5 times in PBT and mounted with VectaShield Plus mounting medium (Vector Laboratories, Burlingame, CA, USA).

Adult brains were prepared following the same protocol of fixation, washing, and DAPI staining used for gut dissection. No antibody staining was performed on adult brains.

### Microscopy and image processing

Images of whole dissected intestines and brains were taken on a Zeiss Axio Zoom microscope. Images of immunostained intestines for quantification were taken on a Zeiss Axio Observer microscope. Image files were prepared using Adobe Photoshop and figures were assembled using Adobe Illustrator.

### Expression pattern and statistical analyses

When screening for EE expression, the numbers of cells expressing *UAS-Stinger* in each of the 11 intestinal subregions (Buchon *et al.* 2013) were scored semiquantitatively from the intestines of five 4- to 8-day-old adult female flies using the method described in Ariyapala *et al.* (2020). When precise cell numbers were determined, 8 intestines were analyzed. When screening for brain expression, brains of five 4- to 8-day-old adult females were dissected and scored.

Statistical analyses were performed in Excel and graphs were made using GraphPad Prism Version 6. For each comparison, midgut regions were evaluated independently using a chi-squared test. Brain data were also analyzed using a chi-squared test.

## Results and discussion

### Screening for split-GAL4 drivers with extensive, enteroendocrine cell-specific expression

To expand the number of split-GAL4 driver combinations useful for EE cell studies, we screened a selection of split-GAL4 drivers using the EE reference drivers *P{R57F07-p65.AD.A}* and *P{R57F07-GAL4.DBD.A}* generated by Ariyapala *et al.* (2020). We will differentiate these drivers from the similar *P{R57F07-p65.AD}* and *P{R57F07-GAL4.DBD}* drivers generated at Janelia Research Campus (Dionne *et al.* 2018) by referring to them as *R57F07.A-p65AD* and *-GAL4DBD.*Supplementary Fig. 1 shows the difference in midgut expression patterns between the *R57F07* and *R57F07.A* split-GAL4 driver pairs, as well as additional characterization of the *R57F07.A* driver pair. To find new EE reference drivers that capture broader EE expression than the *R57F07.A* drivers, we screened drivers from 3 nonexclusive categories (Supplementary Table 2): (1) 42 drivers with enhancer fragments previously reported to direct expression in EEs in either GAL4 or split-GAL4 constructs (Marianes and Spradling 2013; Beehler-Evans and Micchelli 2015; Guo *et al.* 2019; Lim *et al.* 2021); (2) 13 drivers with enhancer fragments found by Ariyapala *et al.* (2020) to direct reporter expression only in EEs (some drivers were characterized previously, while others drivers shared an enhancer fragment with a previously characterized EE driver); and (3) 44 drivers containing enhancers we identified as originating within 10 kb of the genes for the midgut-secreted peptides Allatostatin A, Allatostatin C, Bursicon, CCHamide-1, Diuretic hormone 31, Insulin-like peptide 3, Myoinhibiting peptide precursor, Slit, and Tachykinin (Veenstra *et al.* 2008; Veenstra 2009; Nässel and Winther 2010; Veenstra and Ida 2014; Guo *et al.* 2019; Hung *et al.* 2020; Zhou *et al.* 2020). A total of 41 AD drivers and 47 DBD drivers were screened using the corresponding *R57F07.A* driver and a *UAS-Stinger* reporter. While most of the drivers induced *UAS-Stinger* expression in fewer EE cells than the *R57F07.A* driver pair, we identified a single AD driver that induced expression in a particularly high number of EEs. We present our characterization of this unique driver below.

### Characterization of a novel enteroendocrine cell driver pair

**Fig. 1.**
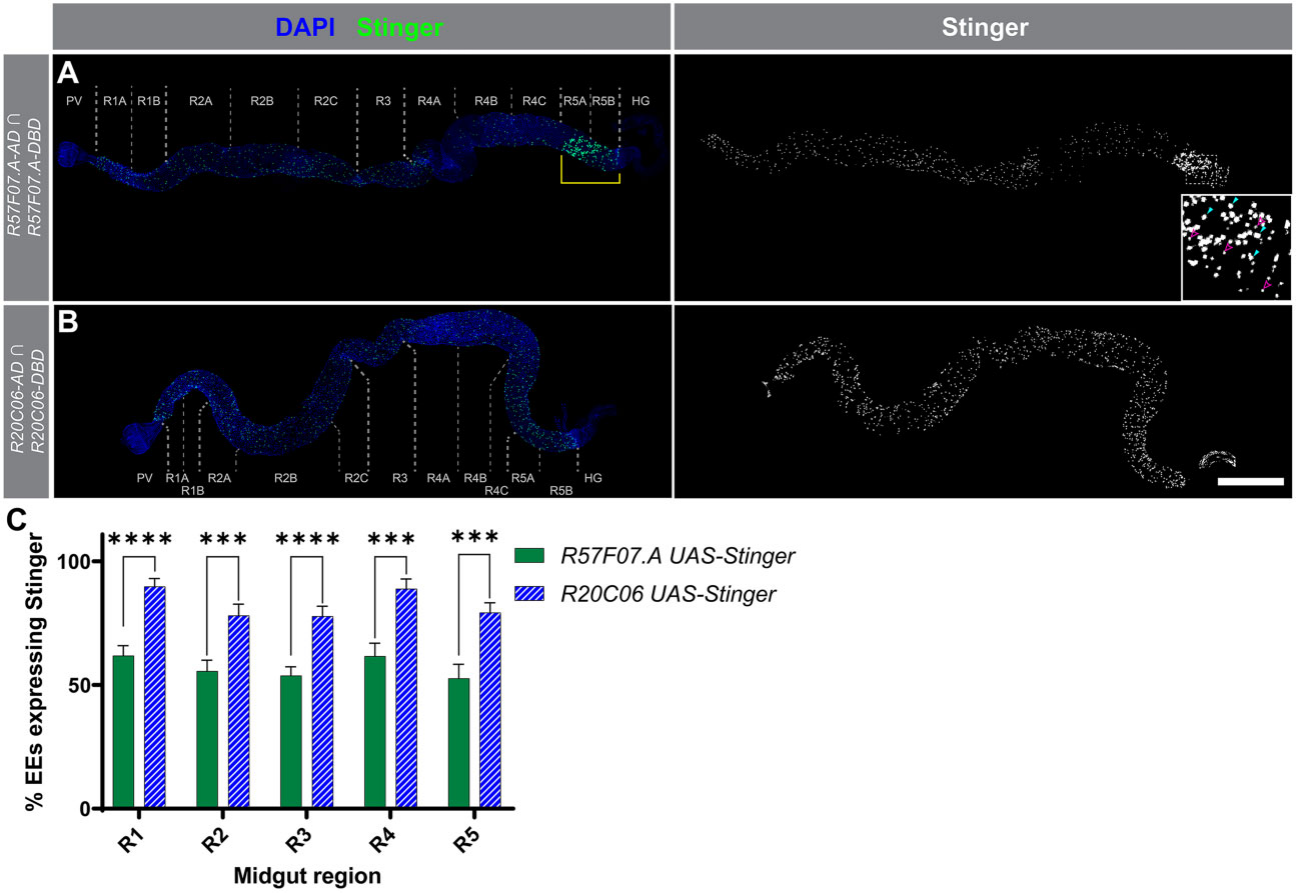
*R20C06* split-GAL4 drivers directed expression in more EEs than *R57F07.A* split-GAL4 drivers. a, b) These micrographs show *UAS-Stinger* expression (right column; superimposed on DAPI nuclear counterstaining in the left column) driven by the *R57F07.A* or *R20C06* split-GAL4 driver pairs. Regional boundaries used in expression pattern analysis throughout the study are indicated. Scale bar 500 µm. a) *R57F07.A* drivers directed expression throughout the gut but drove expression in only ∼60% of EEs. They also drove unexpected expression in large nuclei (likely ECs) in R5 (indicated by bracket). The inset shows an enlargement of the indicated portion of R5, with examples of large nuclei highlighted by filled arrowheads and small nuclei highlighted by open arrowheads. b) *R20C06* drivers directed expression evenly throughout all gut regions and did not drive expression in large nuclei. c) This graph summarizes the percentage of EEs detected by each driver pair and illustrates that, although *R20C06* drivers did not direct expression in all EEs, they directed expression in a significantly larger percentage of EEs than the *R57F07.A* drivers in each midgut region. ***P < 0.001, ****P < 0.0001.

**Fig. 2.**
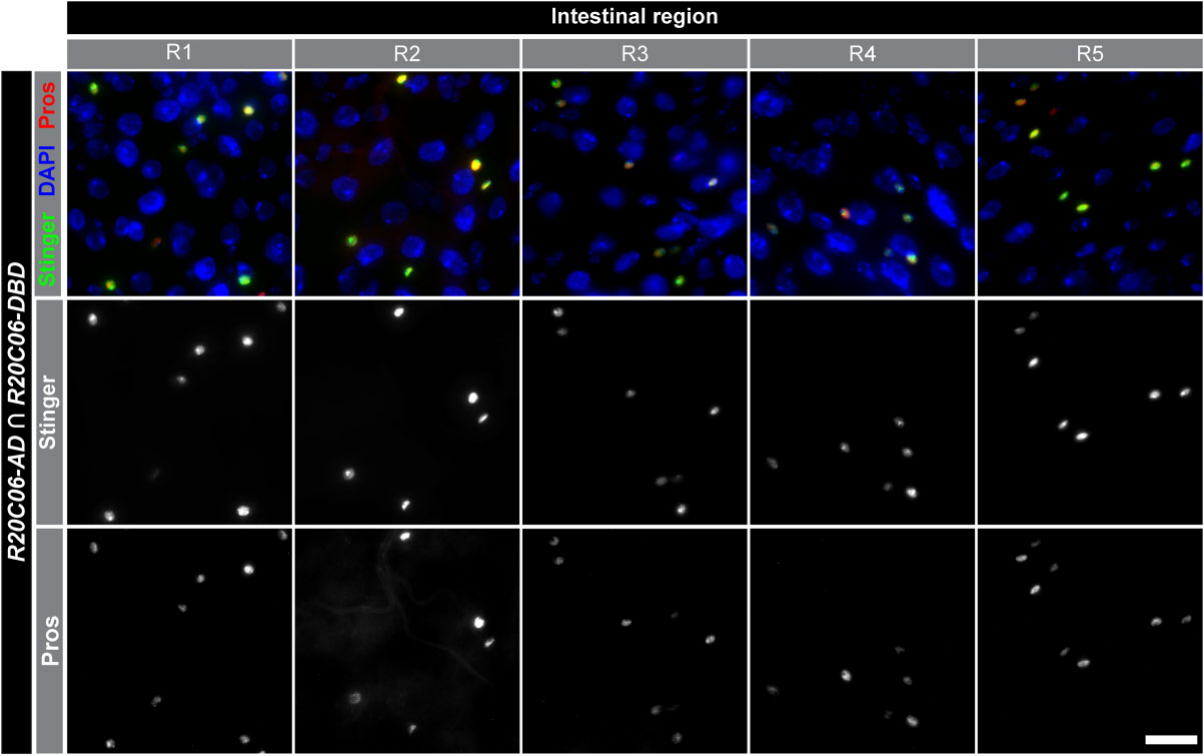
*R20C06* drivers predominantly labeled EE cells. *UAS-Stinger* expression in intestinal regions R1–5 directed by *R20C06* drivers (center row) occurs largely in EEs, which are identified by anti-Pros staining (bottom row). Stinger expression and anti-Prospero staining are shown superimposed on DAPI nuclear counterstaining in the top row. Scale bar 20 µm.

The driver of interest identified by our screen uses the *R20C06* enhancer fragment to direct *p65AD* expression. To evaluate the utility of *R20C06* for detection of EEs, we examined *UAS-Stinger* expression in detail when *R20C06-p65AD* was combined with *R20C06-GAL4DBD*. We observed expression directed by this driver pair in substantially more EEs than induced by the previously characterized *R57F07.A-p65AD* and *-GAL4DBD* driver pair (Ariyapala *et al.* 2020) and the expression was nearly exclusive to EEs based on anti-Pros antibody staining (Figs. 1 and 2). In intestinal regions R1 and R2, no expression driven by the *R20C06* driver pair was observed in other cell types, and R3, R4, and R5 showed non-EE expression in only 2%, 7%, and 2% of Stinger-positive cells, respectively. (These Stinger-positive, Pros-negative cells have small nuclei, which suggests they are a subset of progenitor cells.) *R57F07.A* split-GAL4 drivers together drove no expression in non-EE cells in R2, R3, and R4, and drove expression in non-EEs in 1% of Stinger-positive cells in R1 and 24% in R5, with many of the R5 Stinger-positive, Pros-negative cells having large nuclei (likely ECs). This indicates that both driver pairs conferred quite low levels of *UAS-Stinger* expression in non-EEs, while the *R20C06* pair drove expression in a greater proportion of EEs (approximately 45% more on average). Although some GAL4 drivers with equivalent or superior EE detection capabilities have been identified, such as *P{GawB}pros^V1^* (Scopelliti *et al.* 2014) and *TI{2A-GAL4}CG32547^2A-GAL4^* (Guo *et al.* 2019), this is the most comprehensive detection of EE cells reported to date using any split-GAL4 driver pair.

We examined *R20C06-p65AD* in our screen because, curiously, Ariyapala *et al.* (2020) saw that it drove a distinctly different EE expression pattern than *R20C06-GAL4DBD*, even though these 2 transgenes share an enhancer fragment. They reported no intestinal *UAS* reporter expression in undissected adults when *R20C06-p65AD* was combined with the intestine-specific *CG10116-GAL4DBD* driver, even though *R20C06-GAL4DBD* drove clear *UAS* reporter expression when combined with *CG10116-p65AD*. This unusual difference prompted us to look closely at dissected intestines from flies with *R20C06-p65AD* combined with *CG10116-GAL4DBD*. While we saw a small number of cells in R1a adjacent to the proventriculus expressing *UAS-Stinger* (Supplementary Fig. 2), it is not surprising that expression in so few cells was missed in the previous high-throughput primary screen, which relied on detecting intestinal fluorescence in undissected adults. This pattern is unexpectedly limited, given how extensively these drivers are known to be expressed in EEs from other experiments. It suggests that the expression of one or both drivers is inhibited in this particular genotype, but we have not investigated it further since *R20C06-p65AD* expression appeared otherwise straightforward in the experiments described below. Ariyapala *et al.* (2020) had also shown that the combination of *R20C06-GAL4DBD* and *R57F07.A-p65AD* directed *UAS* reporter expression in EEs but had observed fewer EE cells with expression than we saw with *R20C06-p65AD* and *R57F07.A-GAL4DBD*, particularly in the center of R4. Taken together, these observations show why the *R20C06* drivers were not identified as especially promising EE drivers in the previous study.

**Fig. 3.**
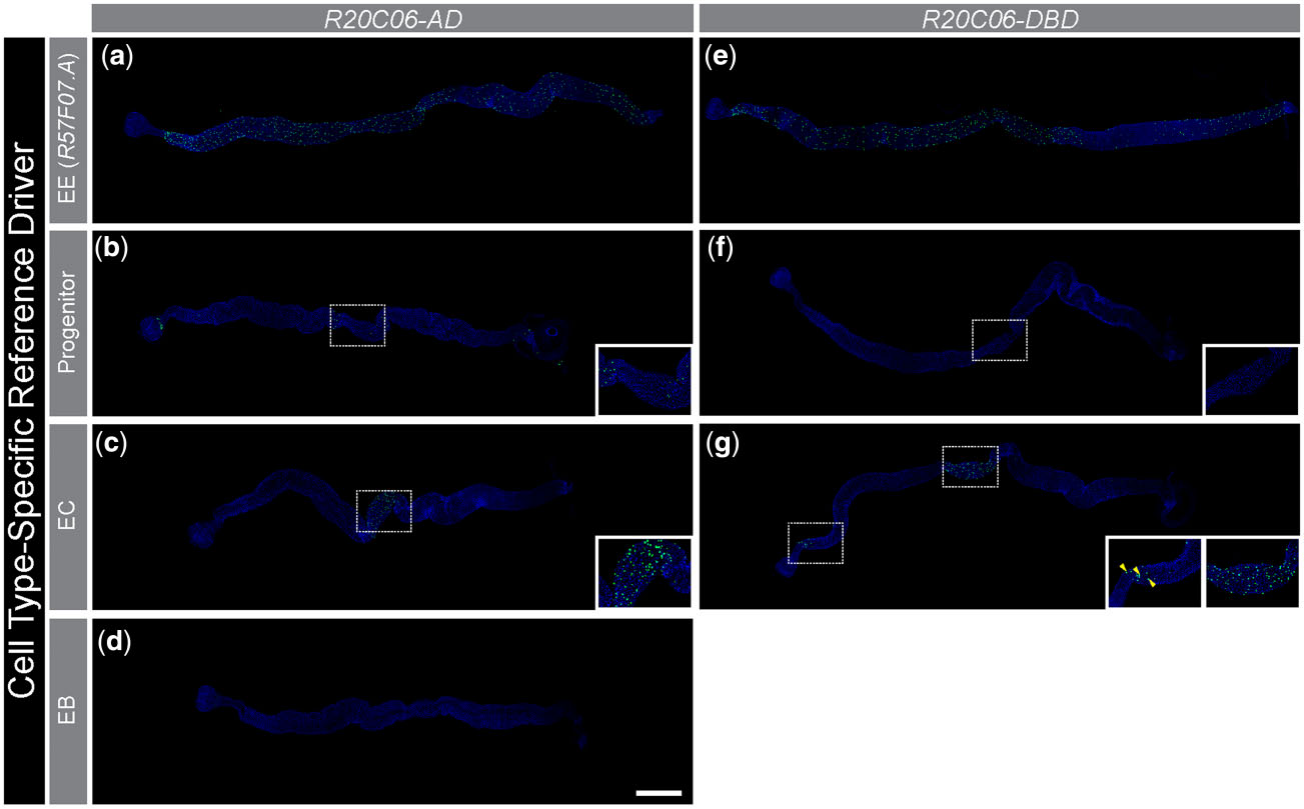
*R20C06* drivers labeled only a few non-EE cells. *R20C06-p65AD* and *R20C06-GAL4DBD* drove intestinal EE expression of *UAS-Stinger* in combination with the *R57F07.A* EE reference drivers (a, e) and drove little to no reporter expression in non-EE cells when combined with progenitor cell (b, f), EC (c, g), or EB (d) reference drivers. Unexpected reporter expression in EEs was seen, primarily in the central midgut, when the *R20C06-p65AD* driver was combined with the *Progenitor-GAL4DBD* driver (b; see Fig. 4 for details), and when either of the *R20C06* drivers was combined with an EC reference driver (c, g right inset; Supplementary Fig. 2). The only instance of unexpected reporter expression involving non-EE cells was observed in large nuclei (likely ECs) when *R20C06-GAL4DBD* was combined with *EC-p65AD* (g left inset, example of large nuclei highlighted by arrowheads). Insets show enlargements of the indicated regions. All intestines are shown with DAPI nuclear counterstaining. Scale bar 500 µm.

To further confirm the EE specificity of the *R20C06-p65AD* and *R20C06-GAL4DBD* drivers, we combined them with the cell-type specific drivers used in Ariyapala *et al.* (2020) for progenitor cells, ECs, and EBs (Fig. 3). The results were consistent with our expectation that these driver pairs would not direct expression in non-EE midgut cells aside from a single exception; namely, that *R20C06-GAL4DBD* drove occasional *UAS-Stinger* expression in large nuclei (likely ECs) when combined with the reference *EC-p65AD* driver. This expression was observed primarily in R1, but it was also seen sporadically in other regions (Fig. 3g). Ariyapala *et al.* (2020) saw *UAS* reporter expression in cells with large nuclei (likely ECs) when several split-GAL4 drivers were combined with the reference *R57F07.A* drivers, including when the *R57F07.A-p65AD* and *-GAL4DBD* reference drivers were combined (also visible in Fig. 1a), so it was not surprising to see similar expression when *R20C06-GAL4DBD* was combined with *EC-p65AD*. Nevertheless, no other driver showed EC-like expression with the *R20C06* drivers (described below), indicating that expression of the *R20C06* drivers was more specific to EEs than expression of the *R57F07.A* drivers.

We also saw *R20C06-p65AD* and *R20C06-GAL4DBD* drivers directing EE expression when combined with the EC reference drivers. In these combinations, we saw *UAS-Stinger* expressed in populations of small cells in R3, particularly near the boundaries with R2 and R4 (Fig. 3c inset and g right inset). We confirmed that these cells were EEs based on anti-Pros antibody staining (Supplementary Fig. 3). Ariyapala *et al.* (2020) saw a similar pattern of expression in EEs in R3 with 25 driver combinations involving the EC reference drivers. Examples of these include *EC-p65AD* combined with *R21H01- GAL4DBD* or *R46F04-GAL4DBD*, as well as *EC-GAL4DBD* combined with *VT021418-p65AD*. These results highlight a minor weakness with the EC reference drivers but are consistent with nearly exclusive EE expression of the *R20C06* drivers.

**Fig. 4.**
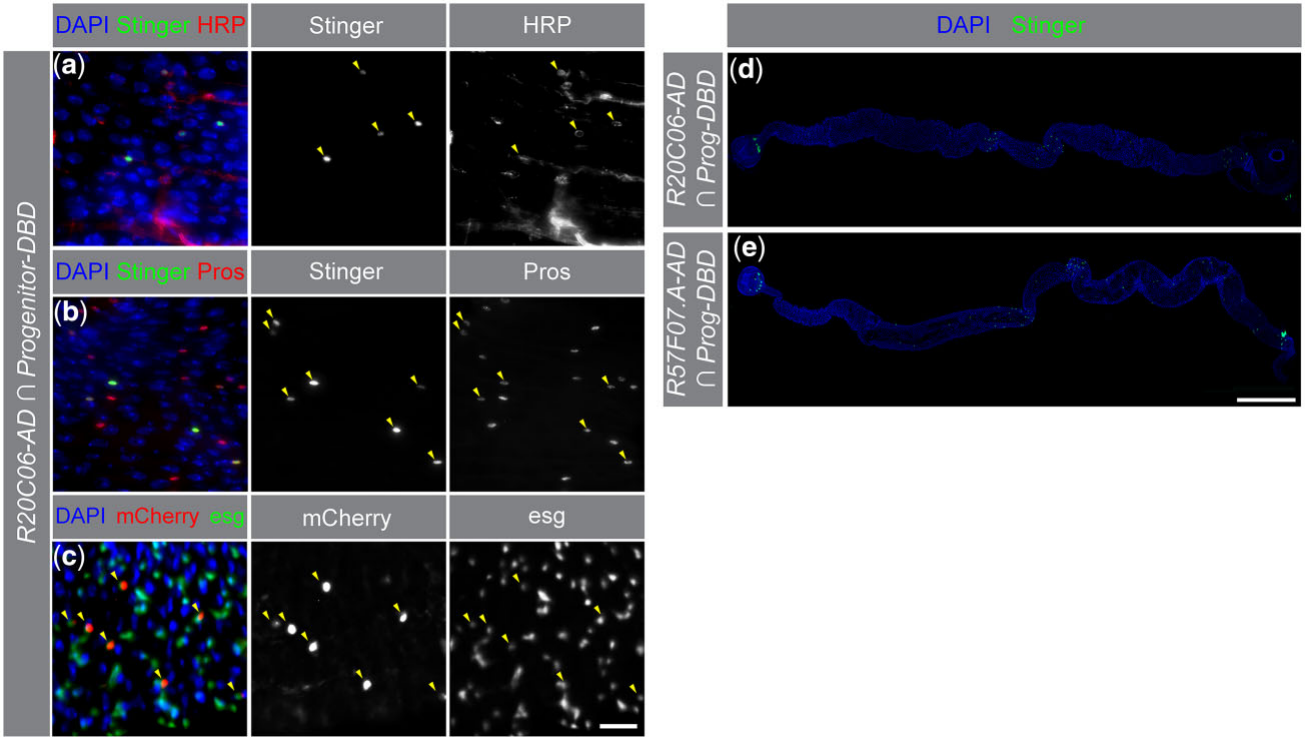
*R20C06-p65AD* drove reporter expression in a unique group of EEs. a–c) When *R20C06-p65AD* was combined with the *Progenitor-GAL4DBD* reference driver and a *UAS* reporter (*UAS-Stinger* or *UAS-mCherry,* center column), the subset of cells that displayed UAS reporter expression also showed staining for both (a) the progenitor cell marker anti-HRP and (b) the EE cell marker anti-Pros (right column), indicating that these cells are part of a unique EE subpopulation identified by Hung *et al.* (2020). These cells also express the progenitor cell marker *TI{sfGFP}esg^KI^*, which places GFP expression under the control of *escargo*t (*esg*) regulatory sequences (c, right column). The left column shows the patterns in the center and right columns superimposed on DAPI nuclear counterstaining. Scale bar 20 µm. (d, e) *R20C06-p65AD* and *R57F07.A-p65AD* drove similar patterns of *UAS-Stinger* expression (superimposed on DAPI nuclear counterstaining) when combined with *Progenitor-GAL4DBD*. *R57F07.A-p65AD* drove additional expression in the proventriculus and the hindgut (e). Scale bar 500 µm.

In addition to the *R20C06* drivers directing *UAS-Stinger* expression in typical EE cells, we saw that *R20C06-p65AD* drove *UAS* reporter expression in EEs at the anterior and posterior boundaries of R3 in combination with the *Progenitor-GAL4DBD* reference driver (Fig. 3b inset). Hung *et al.* (2020) showed that cells in these locations constitute a unique subpopulation of EEs expressing both neuropeptide hormones and genes usually restricted to progenitor cells (but, notably, not *Delta*, the gene most closely associated with progenitor cell identity). Fig. 4, a–c show that all cells identified by these drivers were labeled by both a marker for EE cells, anti-Pros antibody (Shiga *et al.* 1996; Micchelli and Perrimon 2006), and markers for progenitor cells, anti-HRP antibody (O’Brien *et al.* 2011; Miller *et al.* 2020) and a GFP reporter for the *escargot* gene called *TI{sfGFP}esg^KI^* (Micchelli and Perrimon 2006).

In contrast, *R20C06-GAL4DBD* combined with *Progenitor-p65AD* did not direct expression of *UAS-Stinger* in this special EE subpopulation (Fig. 3f inset), a difference that likely reflects the fact that the enhancer fragment in *Progenitor-GAL4DBD* (*VT024642*) is different than that in *Progenitor-p65AD* (*VT004241*). We similarly saw no *UAS-Stinger* expression in these cells when *R57F07.A-GAL4DBD* was combined with *Progenitor-p65AD*, even though we saw expression when *R57F07.A-p65AD* was combined with *Progenitor-GAL4DBD* that was very similar to the expression we saw when we combined *R20C06-p65AD* with *Progenitor-GAL4DBD* (Fig. 4, d and e). Ariyapala *et al.* (2020) reported another driver (*R10F08-p65AD*) that detected this EE subpopulation in combination with the *Progenitor-GAL4DBD* driver as well. All together, these results suggest that the *Progenitor-GAL4DBD* reference driver is a useful tool for marking this subpopulation.

### Reporter effects on expression patterns

**Fig. 5.**
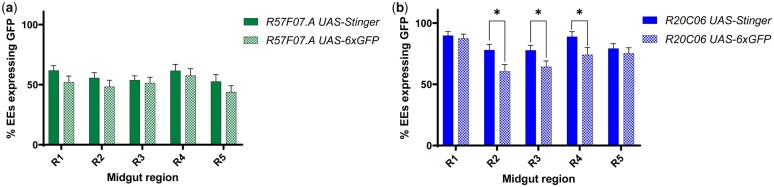
*UAS-Stinger* was a slightly more effective reporter than *UAS-6xGFP* in combination with EE reference drivers. a) When the *R57F07.A* drivers were characterized, there was a slight tendency toward more cells with fluorescence in all midgut regions with a *UAS-Stinger* reporter than a *UAS-6xGFP* reporter. b) This pattern was also observed when characterizing the *R20C06* drivers, with *UAS-Stinger* expressed in significantly more cells than *UAS-6xGFP* in R2, R3, and R4. *P < 0.05.

The EE characterization and screening data reported by Ariyapala *et al.* (2020) were collected using *PBac{UAS-DSCP-6XEGFP}* reporters combined with *R57F07.A* drivers. The extremely bright fluorescence of these *UAS-6xGFP* reporters, arising from 20 copies of *UAS* and 6 copies of the *GFP* coding sequence, was appropriate for that screen but was not necessary for the current study. Instead, we used the *UAS-Stinger* reporters because they are clear and easy to detect without the need for antibody staining yet are not so bright that it is difficult to distinguish individual cells. To evaluate whether these different reporters provided consistent results when combined with the EE reference driver pairs, we examined EE fluorescence when the *R57F07.A* and *R20C06* driver pairs were combined with the *UAS-6xGFP* and *UAS-Stinger* reporters. When both driver pairs were characterized, we saw similar numbers of fluorescent EE cells with *UAS-Stinger* and *UAS-6xGFP* in all midgut regions, but with slightly more fluorescent cells observed with *UAS-Stinger* (Fig. 5). This marginal difference between *UAS-Stinger* and *UAS-6xGFP* expression reached statistical significance only in R2, R3, and R4 when driven by the *R20C06* drivers (Fig. 5b). These results suggest that *UAS-Stinger* reports slightly more comprehensive expression patterns than *UAS-6xGFP* with EE reference drivers.

Furthermore, as described in the previous section, the use of *UAS-Stinger* allowed us to identify an expression pattern resulting from a combination of *R57F07.A* and *Progenitor-GAL4DBD* drivers that had been undetectable using *UAS-6xGFP*. Additional study is necessary to determine the cause of this difference, but it is consistent with previous studies showing that both high levels of p65AD and reporter protein expression from 10 or more *UAS* sites can be toxic to cells (Pfeiffer *et al.* 2010). Taken together, these similarities and differences between *UAS-Stinger* and *UAS-6xGFP* underscore the importance of careful reporter selection and characterization.

### Characterization of the *R20C06* enhancer fragment

**Fig. 6.**
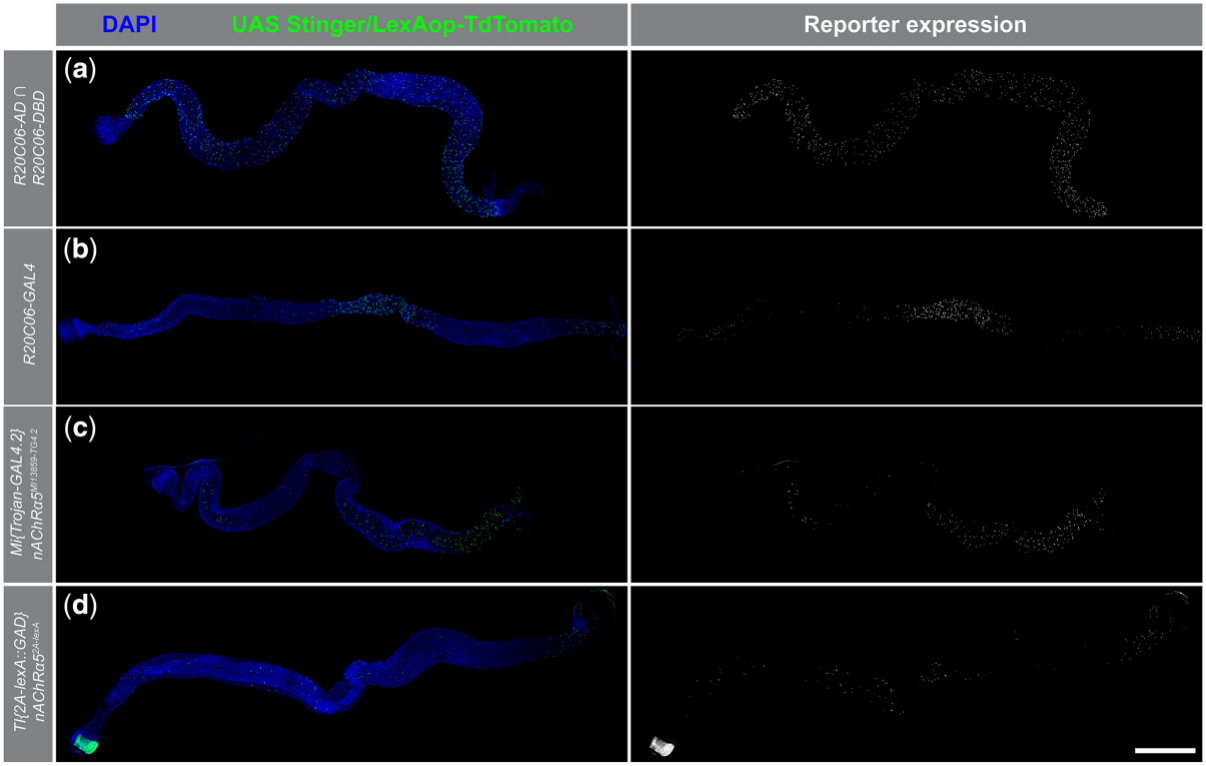
*R20C06* split-GAL4 drivers conferred different expression patterns than the *R20C06-GAL4* driver or *nAChRα5*-associated gene-trap drivers. Split-GAL4 drivers with the *R20C06* enhancer fragment showed expression throughout the entire midgut (a), while the GAL4 driver using the same enhancer fragment showed expression primarily R1, R3, and R5 (b). The *Mi{Trojan-GAL4.2}nAChRα5^MI13859-TG4.2^* GAL4 gene-trap driver showed expression primarily in the posterior midgut (R4 and R5) with additional expression in scattered cells in the anterior and middle midgut (c). The *TI{2A-lexA::GAD}nAChRα5^2A-lexA^* lexA gene-trap driver (d) directed expression similar to that of the *R20C06-GAL4* driver (c), but drove expression in fewer cells. Proventricular expression of *PBac{13XLexAop2-IVS-tdTomato.nls}VK00022* occurs even in the absence of a *lexA* driver (data not shown). *UAS-Stinger* (a–c) and *lexAop-tdTomato.nls* (d) expression are shown in the right column, and superimposed on nuclear DAPI staining in the left column. Scale bar 500 µm.

In an attempt to identify additional tools useful for the study of EEs, we examined other drivers and cell markers related to the *R20C06* enhancer fragment. We compared the midgut expression pattern of the split-GAL4 *R20C06* driver pair to that of the GAL4 driver containing the same enhancer fragment using a *UAS-Stinger* reporter (Fig. 6, a and b). The GAL4 driver showed a notably more restricted expression pattern than the split-GAL4 pair: the GAL4 driver displayed very little expression in R2 and R4 while the split-GAL4 pair showed evenly distributed expression through all 5 of the major midgut regions. This is not entirely surprising, as idiosyncratic differences between GAL4 and split-GAL4 drivers have been observed in other instances as well (Luan *et al.* 2006; Pfeiffer *et al.* 2010; Ariyapala *et al.* 2020). Pfeiffer *et al.* (2010) suggested that split-GAL4 drivers may direct more widespread expression than their cognate GAL4 drivers due to the use of the stronger p65 activation domain. Regardless, these results indicate that *R20C06-GAL4* is not as well suited for use as a pan-EE driver as the corresponding split-GAL4 drivers.

The *R20C06* enhancer fragment lies within the *nicotinic Acetylcholine Receptor α5* (*nAChRα5*) gene and there are no other genes within 16 kb of this fragment (Larkin *et al.* 2021). To investigate possible relationships between the midgut expression patterns of the *R20C06* drivers and *nAChRα5*, we compared driver expression to relevant intestinal RNAseq data (Guo *et al.* 2019; Hung *et al.* 2020) and to the expression of protein-trap and gene-trap insertions associated with *nAChRα5*. RNAseq data shows that *nAChRα5* is expressed in a variety of EE types throughout the gut (Guo *et al.* 2019) and that the gene is expressed in the largest number of cells in R4 and R5 (Dutta *et al.* 2013). When we examined the expression pattern of *Mi{Trojan-GAL4.2}nAChRα5^MI13859-TG4.2^*, a gene trap for *nAChRα5*, with a *UAS-Stinger* reporter, we saw expression that corresponded to the RNAseq data: expression in the greatest number of cells in the posterior midgut with additional expression in fewer cells in the anterior midgut (Fig. 6c). When we examined *lexAop-tdTomato.nls* expression driven by *TI{2A-lexA::GAD}nAChRα5^2A-lexA^*, a gene-trap insertion expressing lexA (Fig. 6d), we observed a similar pattern to that of *R20C06-GAL4*, but with expression in fewer cells. In addition, we analyzed the *nAChRα5* protein-trap insertion *Mi{PT-GFSTF.0}nAChRα5^MI05549-GFSTF.0^*, but we were not able to detect any expression in the midgut epithelium using immunostaining against GFP (data not shown). Based on these observations, the *R20C06* split-GAL4 drivers behave differently than GAL4 drivers containing the same enhancer fragment and the protein and gene traps for the nearby *nAChRα5* gene. Therefore, these other genetic elements do not present promising avenues for future pan-EE tool development. More broadly speaking, these results reemphasize that isolated enhancers or enhancer fragments are often not sufficient to recapitulate the native expression of genes and that the expression patterns driven by enhancers also depend on adjacent sequences and genomic contexts.

### Other split-GAL4 drivers that detect enteroendocrine cells in the Drosophila midgut

During the screen that identified the *R20C06* split-GAL4 driver pair, we collected expression pattern data for all the split-GAL4 drivers screened with *R57F07.A*. We later combined all drivers from that initial screen with the *R20C06* drivers so that we could evaluate differences and similarities in expression patterns and explore the utility of the *R20C06* driver pair for EE detection in the split-GAL4 system. Overall, 88 split-GAL4 drivers were tested. There were 42 drivers (48%) positive for adult midgut expression when crossed to the *R57F07.A* drivers and 39 drivers (44%) positive when crossed to the *R20C06* drivers (Supplementary Table 3). All expression pattern data from these screens are available at https://bdsc.indiana.edu/stocks/gal4/midgut_EEs.html.

**Fig. 7.**
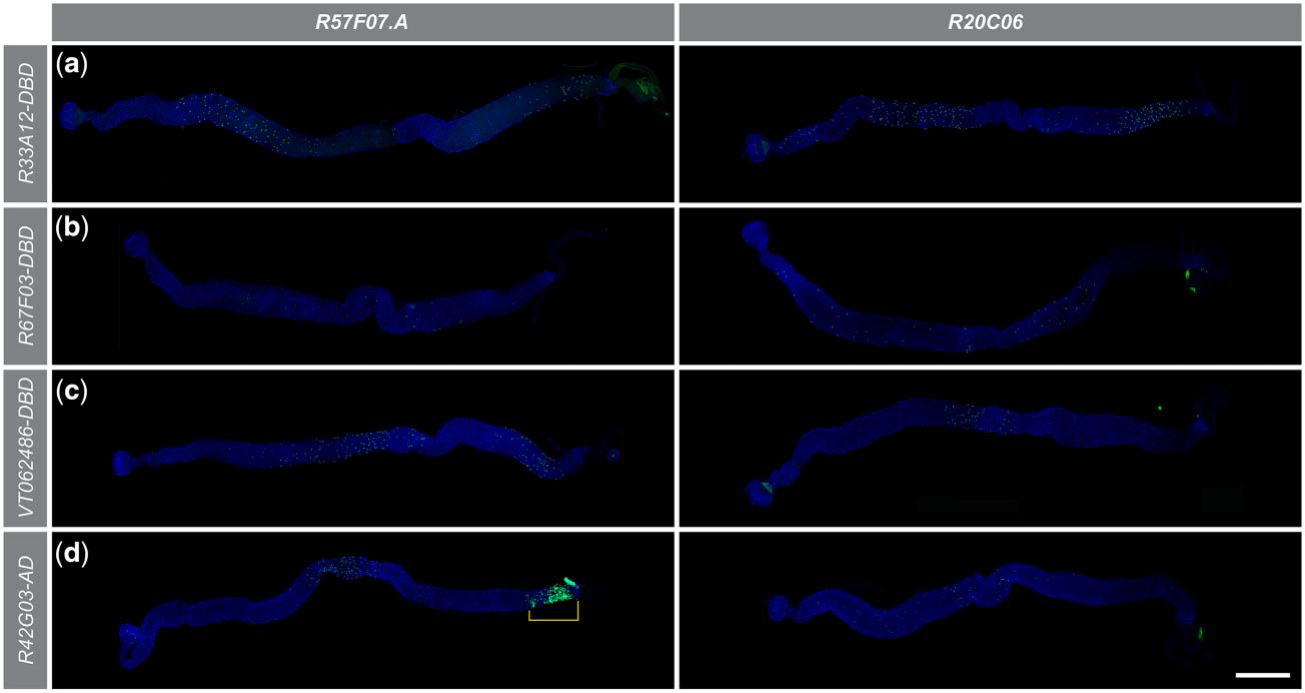
*R57F07.A* and *R20C06* reference drivers directed different expression patterns when combined with the same experimental split-GAL4 driver. These micrographs show representative examples of *UAS-Stinger* expression (green) when split-GAL4 drivers were combined with the corresponding *R57F07.A* (left) or *R20C06* (right) split-GAL4 driver. a) *R33A12-GAL4DBD* drove similar patterns when combined with both reference drivers. b) *R67F03-GAL4DBD* drove more expression when combined with *R20C06-p65AD* than with *R57F07.A-p65AD*. c) *VT062486-GAL4DBD* drove more expression when combined with *R57F07.A-p65AD* than with *R20C06-p65AD*. d) *R42G03-p65AD* drove similar expression patterns when combined with both reference drivers, but also drove expression in large nuclei (likely ECs) with *R57F07.A-GAL4DBD* as indicated by bracket. Scale bar 500 µm.

There were 3 drivers (*R75B09-GAL4DBD*, *R47G08-GAL4DBD*, *R27B07-GAL4DBD*) which activated *UAS-Stinger* expression when crossed to an *R57F07.A* driver but not when crossed to a *R20C06* driver, while only one driver (*R65D06-GAL4DBD*) activated *UAS-Stinger* expression when crossed to a *R20C06* driver but not when crossed to a *R57F07.A* driver. Drivers expressing with both *R57F07.A* and *R20C06* fell into 3 classes: (1) 64 drivers showed similar patterns when crossed to either reference driver (Fig. 7a); (2) 12 drivers gave expression in more cells with the *R20C06* than the *R57F07.A* reference driver (Fig. 7b); and (3) 7 drivers gave expression in more cells with the *R57F07.A* than the *R20C06* reference driver (Fig. 7c). It is important to note that even if a driver is in category 1, more research is necessary to verify that identical cell populations are being identified by both drivers. In addition, crosses with *R20C06* did not show any expression in big nuclei (likely ECs), while this was somewhat common in crosses with *R57F07.A* (e.g. R5 in Fig. 7d). These results indicated that, overall, both *R57F07.A* and *R20C06* drivers detected many EEs in the adult midgut. The *R57F07.A* drivers directed expression in a few situations that *R20C06* drivers did not, but the *R20C06* drivers conferred other advantages, such as less non-EE expression and more combinations expressing in a greater number of EEs.

**Fig. 8.**
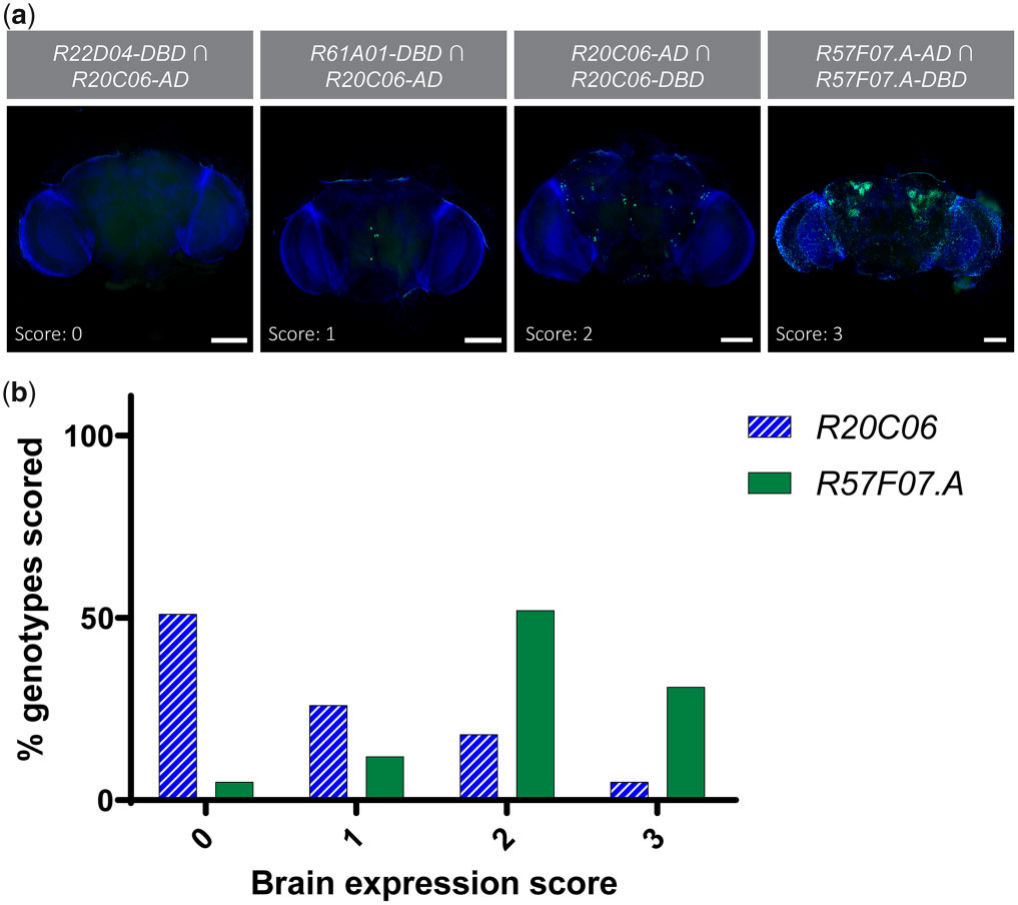
*R20C06* drivers direct less expression in the brain than *R57F07.A* drivers. a) Representative examples of each score are shown, with *UAS-Stinger* expression shown superimposed on nuclear DAPI counterstaining. Brains were scored on a scale of 0 to 3, where 0 indicated no clear reporter expression, 1 indicated a few (≤9) cells with expression, 2 indicated a moderate number of cells (10–60) with expression, and 3 indicated many cells (>60) with expression. Scale bars 100 µm. b) The 36 split-GAL4 combinations involving *R20C06* drivers showed a significantly stronger propensity toward lower scores than the 42 combinations involving *R57F07.A* drivers (*P* < 0.001).

We also examined adult brains from split-GAL4 combinations that drove expression in EEs. Because brains were dissected only in cases where midgut *UAS-Stinger* expression was present, the total number of genotypes with brains scored varied slightly between *R20C06* and *R57F07.A* drivers. We scored brains from 39 driver pairs using *R20C06* and 42 driver pairs using *R57F07.A* on a semiquantitative scale from 0–3, with 0 indicating no expression and 3 indicating a large number of cells with expression (Fig. 8a). When we analyzed brains from progeny of *R57F07.A* crosses, 17% of genotypes examined had a score of 1 or 0 (little to no expression), while 77% of genotypes from crosses involving *R20C06* drivers had a score of 1 or 0. Conversely, only 5% of genotypes with *R20C06* drivers received a score of 3 (large number of cells with expression) whereas 31% genotypes with *R57F07.A* drivers had a score of 3 (Fig. 8b, Supplementary Table 3). These results demonstrated that *R20C06* drivers directed significantly less reporter expression in the brain, which suggests that these drivers are more useful tools than *R57F07.A* drivers for disentangling the physiological roles of hormone-secreting cells in the gut from those secreting the same peptides in the brain.

In summary, combinations of experimental drivers and the 2 corresponding reference drivers can, in some cases, drive distinctly different EE expression patterns. The differences between the *R20C06* and *R57F07.A* drivers expand available options and allow researchers to select the reference drivers more suitable to a given EE population of interest. Further research is necessary to characterize these EE-specific reference drivers more deeply, understand their similarities and differences in greater detail, and elaborate on the advantages unique to each driver pair.

## Data availability

The accompanying tables contain complete characterization data. Supplementary Table 1 contains antibody details and genotypes for each strain used. Supplementary Table 2 provides the rationale for inclusion of drivers in the screen. Supplementary Table 3 contains characterization data collected in the screen. Supplementary Table 4 lists the genotypes of all strains shown in figures. Intestine images and screen data are available at https://bdsc.indiana.edu/stocks/gal4/midgut_EEs.html. Extant stocks are available from the Bloomington Drosophila Stock Center (https://bdsc.indiana.edu) or upon request.

Supplemental material is available at *G3* online.

## Supplementary Material

jkac102_Supplemental_Figure_Caption

jkac102_Supplemental_Figure_S1

jkac102_Supplemental_Figure_S2

jkac102_Supplemental_Figure_S3

jkac102_Supplemental_Table_S1

jkac102_Supplemental_Table_S2

jkac102_Supplemental_Table_S3

jkac102_Supplemental_Table_S4

## References

[jkac102-B1] Ariyapala IS , Holsopple JM, Popodi EM, Hartwick DG, Kahsai L, Cook KR, Sokol NS. Identification of split-GAL4 drivers and enhancers that allow regional cell type manipulations of the *Drosophila melanogaster* intestine. Genetics. 2020;216(4):891–903. 32988987 10.1534/genetics.120.303625 PMC7768249

[jkac102-B2] Beehler-Evans R , Micchelli CA. Generation of enteroendocrine cell diversity in midgut stem cell lineages. Development. 2015;142(4):654–664. 25670792 10.1242/dev.114959 PMC4325375

[jkac102-B3] Buchon N , Osman D, David FPA, Fang HY, Boquete J-P, Deplancke B, Lemaitre B. Morphological and molecular characterization of adult midgut compartmentalization in Drosophila. Cell Rep. 2013;3(5):1725–1738. 23643535 10.1016/j.celrep.2013.04.001

[jkac102-B4] Dionne H , Hibbard KL, Cavallaro A, Kao JC, Rubin GM. Genetic reagents for making split-GAL4 lines in Drosophila. Genetics. 2018;209(1):31–35. 29535151 10.1534/genetics.118.300682 PMC5937193

[jkac102-B5] Dutta D , Xiang J, Edgar BA. RNA expression profiling from UNIT 2F.2 FACS-isolated cells of the Drosophila intestine. Curr Protoc Stem Cell Biol. 2013;1:2F.2.1–2F.2.12. 10.1002/9780470151808.sc02f02s27 24510286

[jkac102-B6] Guo X , Yin C, Yang F, Zhang Y, Huang H, Wang J, Deng B, Cai T, Rao Y, Xi R, et al The cellular diversity and transcription factor code of Drosophila enteroendocrine cells. Cell Rep. 2019;29(12):4172–4185. 31851941 10.1016/j.celrep.2019.11.048

[jkac102-B7] Hung R-J , Hu Y, Kirchner R, Liu Y, Xu C, Comjean A, Tattikota SG, Li F, Song W, Ho Sui S, et al A cell atlas of the adult Drosophila midgut. Proc Natl Acad Sci USA. 2020;117(3):1514–1523. 31915294 10.1073/pnas.1916820117 PMC6983450

[jkac102-B8] Larkin A , Marygold SJ, Antonazzo G, Attrill H, Dos Santos G, Garapati PV, Goodman JL, Gramates LS, Millburn G, Strelets VB, et al; FlyBase Consortium. FlyBase: updates to the *Drosophila melanogaster* knowledge base. Nucleic Acids Res. 2021;49(D1):D899–D907. 33219682 10.1093/nar/gkaa1026 PMC7779046

[jkac102-B9] Lim SY , You H, Lee J, Lee J, Lee Y, Lee K-A, Kim B, Lee J-H, Jeong J, Jang S, et al Identification and characterization of GAL4 drivers that mark distinct cell types and regions in the Drosophila adult gut. J Neurogenet. 2021;35(1):33–44. 33326321 10.1080/01677063.2020.1853722

[jkac102-B10] Luan H , Peabody NC, Vinson CRR, White BH. Refined spatial manipulation of neuronal function by combinatorial restriction of transgene expression. Neuron. 2006;52(3):425–436. 17088209 10.1016/j.neuron.2006.08.028 PMC1713190

[jkac102-B11] Marianes A , Spradling AC. Physiological and stem cell compartmentalization within the Drosophila midgut. Elife. 2013;2013:1–19. 10.7554/eLife.00886 PMC3755342 23991285

[jkac102-B12] Micchelli CA , Perrimon N. Evidence that stem cells reside in the adult Drosophila midgut epithelium. Nature. 2006;439(7075):475–479. 16340959 10.1038/nature04371

[jkac102-B13] Miguel-Aliaga I , Jasper H, Lemaitre B. Anatomy and physiology of the digestive tract of *Drosophila melanogaster*. Genetics. 2018;210(2):357–396. 30287514 10.1534/genetics.118.300224 PMC6216580

[jkac102-B14] Miller DE , Kahsai L, Buddika K, Dixon MJ, Kim BY, Calvi BR, Sokol NS, Hawley RS, Cook KR. Identification and characterization of breakpoints and mutations on *Drosophila melanogaster* balancer chromosomes. G3 (Bethesda). 2020;10(11):4271–4285. 32972999 10.1534/g3.120.401559 PMC7642927

[jkac102-B15] Nässel DR , Winther ÅME. Drosophila neuropeptides in regulation of physiology and behavior. Prog Neurobiol. 2010;92(1):42–104. 20447440 10.1016/j.pneurobio.2010.04.010

[jkac102-B16] O'Brien LE , Soliman SS, Li X, Bilder D. Altered modes of stem cell division drive adaptive intestinal growth. Cell. 2011;147(3):603–614. 22036568 10.1016/j.cell.2011.08.048 PMC3246009

[jkac102-B17] Ohlstein B , Spradling A. The adult Drosophila posterior midgut is maintained by pluripotent stem cells. Nature. 2006;439(7075):470–474. 16340960 10.1038/nature04333

[jkac102-B18] Pfeiffer BD , Ngo T-TB, Hibbard KL, Murphy C, Jenett A, Truman JW, Rubin GM. Refinement of tools for targeted gene expression in Drosophila. Genetics. 2010;186(2):735–755. 20697123 10.1534/genetics.110.119917 PMC2942869

[jkac102-B19] Scopelliti A , Cordero JB, Diao F, Strathdee K, White BH, Sansom OJ, Vidal M. Article local control of intestinal stem cell homeostasis by enteroendocrine cells in the adult Drosophila midgut. Curr Biol. 2014;24(11):1199–1211. 24814146 10.1016/j.cub.2014.04.007 PMC4046228

[jkac102-B20] Shiga Y , Tanaka-Matakatsu M, Hayashi S. A nuclear GFP/beta-galactosidase fusion protein as a marker for morphogenesis in living Drosophila. Dev Growth Differ. 1996;38(1):99–106.

[jkac102-B21] Veenstra JA. Peptidergic paracrine and endocrine cells in the midgut of the fruit fly maggot. Cell Tissue Res. 2009;336(2):309–323. 19319573 10.1007/s00441-009-0769-y

[jkac102-B22] Veenstra JA , Agricola HJ, Sellami A. Regulatory peptides in fruit fly midgut. Cell Tissue Res. 2008;334(3):499–516. 18972134 10.1007/s00441-008-0708-3

[jkac102-B23] Veenstra JA , Ida T. More Drosophila enteroendocrine peptides: orcokinin B and the CCHamides 1 and 2. Cell Tissue Res. 2014;357(3):607–621. 24850274 10.1007/s00441-014-1880-2

[jkac102-B24] Zhou X , Ding G, Li J, Xiang X, Rushworth E, Song W. Physiological and pathological regulation of peripheral metabolism by gut-peptide hormones in Drosophila. Front Physiol. 2020;11:577717. 33117196 10.3389/fphys.2020.577717 PMC7552570

[jkac102-B25] Zwick RK , Ohlstein B, Klein OD. Intestinal renewal across the animal kingdom: comparing stem cell activity in mouse and Drosophila. Am J Physiol Gastrointest Liver Physiol. 2019;316(3):G313–G322. 30543448 10.1152/ajpgi.00353.2018 PMC6415738

