# Supplementary material for: Identification of novel split-GAL4 drivers for the characterization of enteroendocrine cells in the Drosophila melanogaster midgut
Source: G3 (Bethesda). 2022 Apr 29;12(6):jkac102. doi: 10.1093/g3journal/jkac102 (PMC9157172; doi:10.1093/g3journal/jkac102)
Supplement: jkac102_Supplemental_Figure_Caption [file jkac102_supplemental_figure_caption.docx]

Figure S1. Characterizing split-GAL4 drivers with the *R57F07.A* enhancer fragment. The *P{R57F07-p65.AD.A}* and *P{R57F07-GAL4.DBD.A}* drivers made by Ariyapala *et al.* (2020) (A) direct a different and much more expansive pattern of *UAS-Stinger* expression (green in left column, white in right column) than the *P{R57F07-p65.AD}* and *P{R57F07-GAL4.DBD}* drivers made by Dionne *et al.* (2018) (B) containing the same enhancer fragment. The *R57F07.A* drivers conferred Stinger expression throughout all midgut regions (A), while the *R57F07* drivers conferred expression largely limited to the posterior midgut (B). Scale bar 500 µm. (C) In this study, we observed different reporter expression in EEs with the *R57F07.A* drivers than previously reported, although this difference was restricted to just one region. As shown in the graph, Ariyapala *et al.* (2020) reported low expression in R4, while we have consistently seen uniform expression across all midgut regions. This discrepancy is not explained by differences in reporter insertions as the third chromosome *P{UAS-DSCP-6XEGFP}attP2* reporter used by Ariyapala *et al.* (2020) gave us the same expression in R4 as the second chromosome *PBac{UAS-DSCP-6XEGFP}VK00018* reporter shown here. ****p<0.0001.

Figure S2. *R20C06-p65AD* showed minimal expression when combined with the intestine-specific *CG10116-GAL4DBD* driver. *UAS-Stinger* is shown in green and nuclear DAPI staining is shown in blue. The arrowhead indicates the only segment of the midgut where reporter expression was seen. Expression was typically present in ~5 cells. Scale bar 500 µm.

Figure S3. Reporter expression was seen in enteroendocrine cells of R3 when *R20C06* drivers were combined with EC reference drivers. Unexpected *UAS-Stinger* expression (green in panels A, D, white in panels B, E) was observed in R3 when *R20C06-p65AD* was combined with *EC-GAL4DBD* and when *R20C06-GAL4DBD* was combined with *EC-p65AD.* Anti-Prospero antibody staining (red in panels A, D, white in panels C, F) showed that this *UAS-Stinger* expression occurred in enteroendocrine cells. In addition, *UAS-Stinger* expression was observed in some non-EE cells (likely ECs), primarily in R1, when *R20C06-GAL4DBD* was combined with *EC-p65AD* (not seen here, but shown in Figure 3G). All nuclei were stained with DAPI, shown in blue in panels A and D. Scale bar 40 µm.
