# Supplementary figures and images for: Identification of novel split-GAL4 drivers for the characterization of enteroendocrine cells in the Drosophila melanogaster midgut
Source: G3 (Bethesda). 2022 Apr 29;12(6):jkac102. doi: 10.1093/g3journal/jkac102 (PMC9157172; doi:10.1093/g3journal/jkac102)

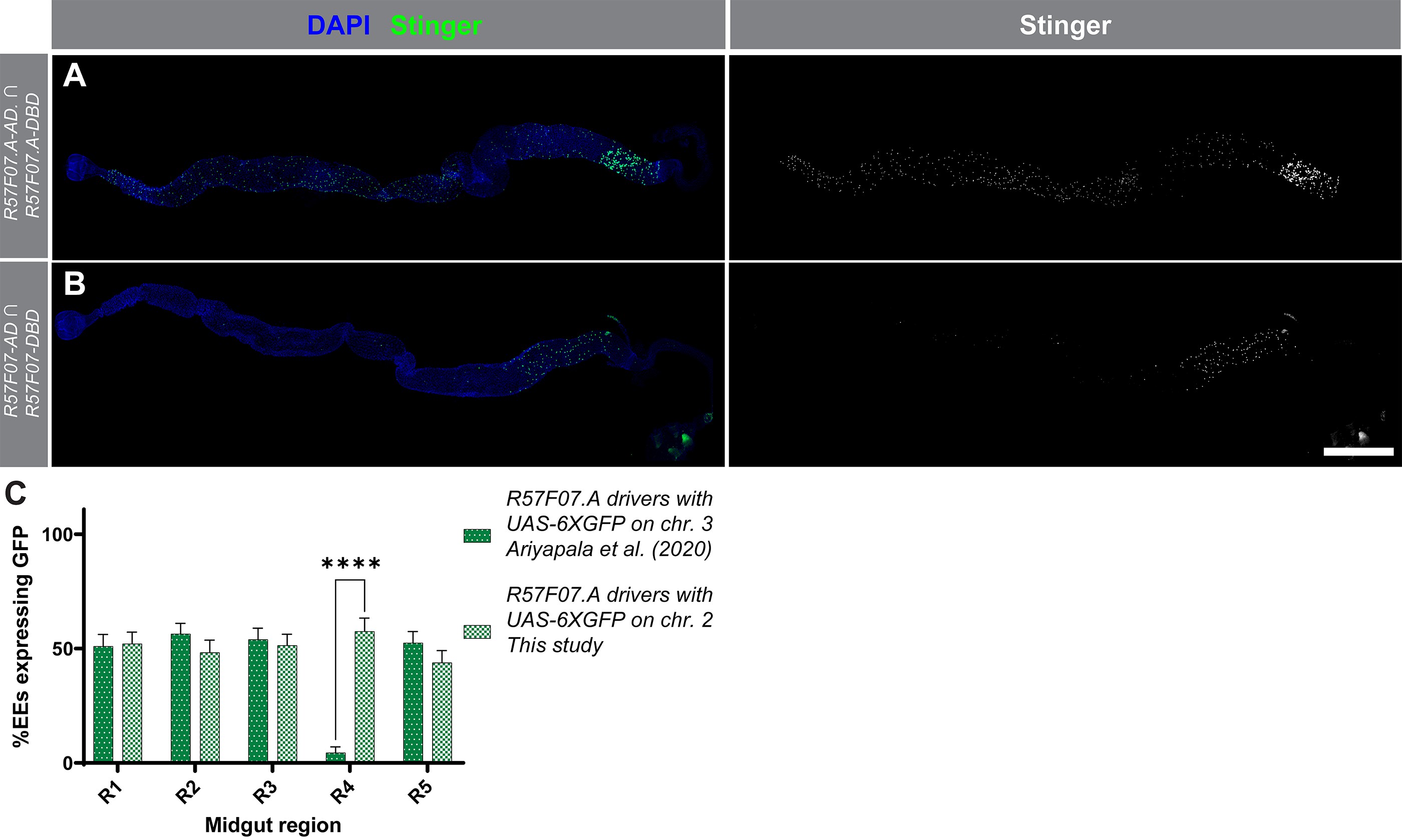

Supplement: jkac102_Supplemental_Figure_S1 [file jkac102_supplemental_figure_s1.jpeg]

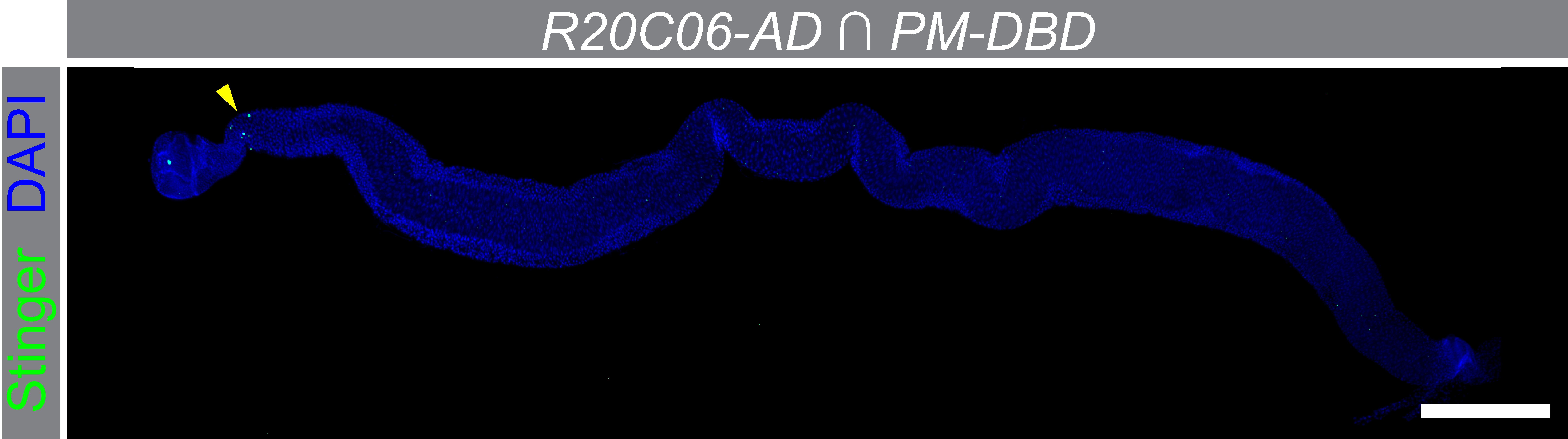

Supplement: jkac102_Supplemental_Figure_S2 [file jkac102_supplemental_figure_s2.jpeg]

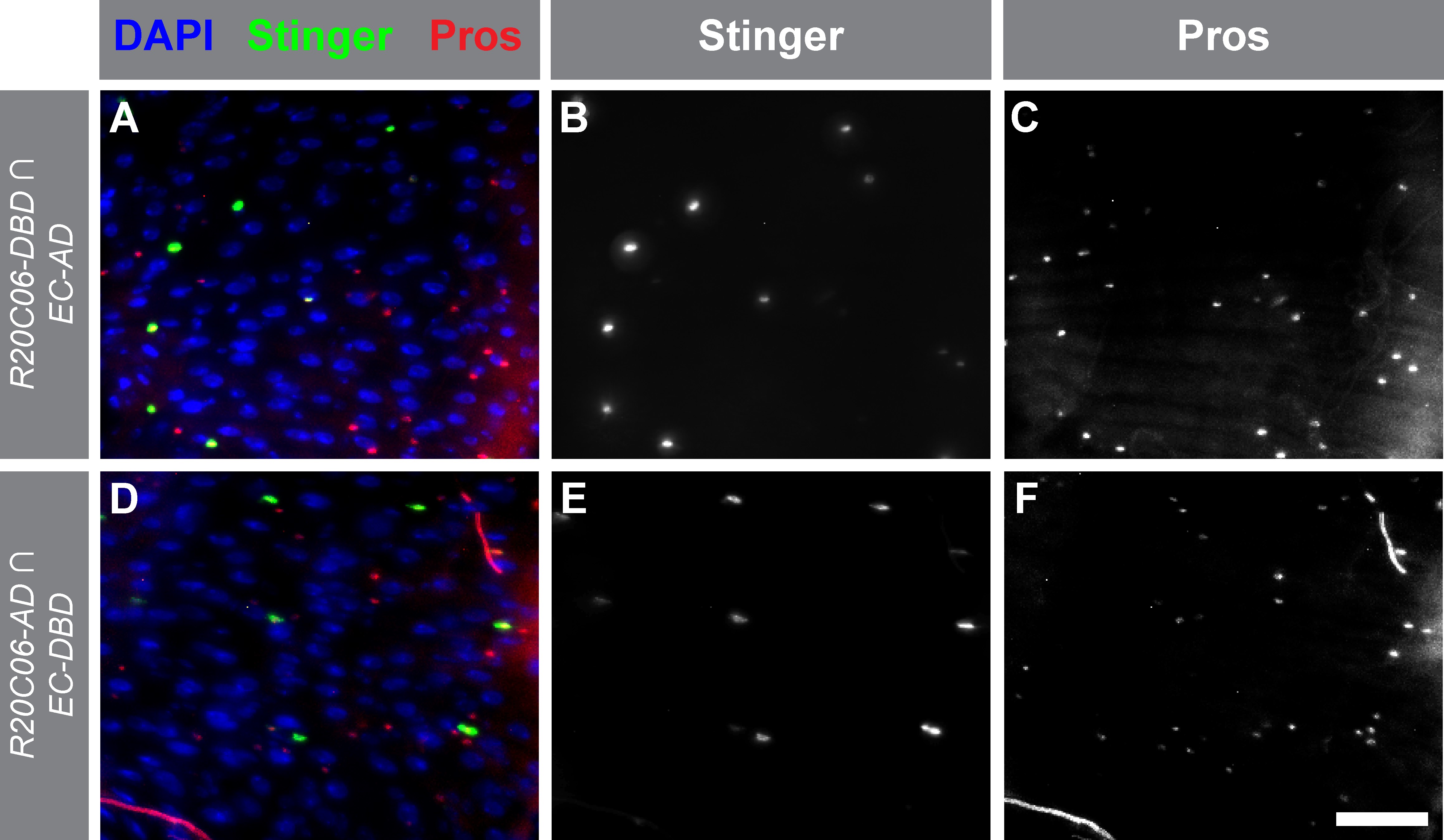

Supplement: jkac102_Supplemental_Figure_S3 [file jkac102_supplemental_figure_s3.jpeg]
